# Dermatophytes and Dermatophytosis in Cluj-Napoca, Romania—A 4-Year Cross-Sectional Study

**DOI:** 10.3390/jof6030154

**Published:** 2020-08-28

**Authors:** Ioana Alina Colosi, Odile Cognet, Horațiu Alexandru Colosi, Marcela Sabou, Carmen Costache

**Affiliations:** 1 Department of Microbiology, Iuliu Hațieganu University of Medicine and Pharmacy, 400349 Cluj-Napoca, Romania; icolosi@umfcluj.ro (I.A.C.); carmen_costache@yahoo.com (C.C.); 2 Department of Laboratory Medicine, Regina Maria Private Health Network, Unirea Medical Center, 400117 Cluj-Napoca, Romania; 3 Parasitology-Mycology Laboratory, Grenoble Alpes University Hospital, 38043 Grenoble CEDEX 9, France; ofaure@chu-grenoble.fr; 4 Division of Medical Informatics and Biostatistics, Department of Medical Education, Iuliu Hațieganu University of Medicine and Pharmacy, 400349 Cluj-Napoca, Romania; 5 Dynamique des Interactions Hôte-Pathogène UR 7292, Université de Strasbourg, F-67000 Strasbourg, France; amsabou@unistra.fr

**Keywords:** dermatophytes, epidemiology, dermatophytosis, diagnosis, molds, yeasts

## Abstract

Dermatophytes are filamentous keratinophilic fungi which affect nails, skin, and hair. Their variable distribution in the world justifies local epidemiological studies. During recent decades, few studies have been published regarding the epidemiology and etiology of dermatophytosis in Romania. The aim of this study was to identify the dermatophytes isolated from superficial fungal infections. To the best of our knowledge, this is the first such study conducted in the area of North-Western Romania. Over the past four years, samples collected from outpatients with suggestive lesions for dermatophytoses (nails, skin, hair), who addressed several private practice dermatologists from Cluj-Napoca, Romania, were sent to a specialized laboratory and examined by microscopy and culture. A total of 350 samples from 322 patients were examined. One hundred samples (28.6%) collected from 90 patients (27.9%) were positive by direct microscopy and/or culture. Among the 63 positive cultures (18%), 44 dermatophytes (69.8%), 2 molds (3.2%), and 17 yeasts (27%) were isolated. The main dermatophyte species identified were *Trichophyton rubrum* (mostly from onychomycosis) and *Microsporum canis* (from tinea capitis and tinea corporis in children). Yeasts (*Candida* species) were isolated from nails, especially from women.

## 1. Introduction

Dermatophytes are filamentous keratinophylic fungi from the genera *Trichophyton*, *Microsporum*, *Epidermophyton*, and *Nannizzia*, which affect nails, skin, and hair. Dermatophytoses are fungal infections that are frequently diagnosed all over the world. These infections are usually not severe, but can sometimes be rather difficult to treat, exhibiting a chronic evolution [1,2,3,4,5,6,7,8]. Anthropophilic, zoophilic, and geophilic sources are known for various dermatophytes. The widespread but uneven distribution of dermatophytes in the world justifies the need for local epidemiological studies [9,10], as it is essential to identify the precise etiological agents for the effective management, treatment, and prevention of dermatophytosis [11,12,13]. During recent decades, insufficient studies have been performed and published regarding the ecology, epidemiology, and etiology of dermatophytosis in Romania. Very little information is available about the etiology of superficial fungal infections in Romania: several old studies, performed between 1968 and 1973 in Bucharest (South-Eastern Romania) [14,15,16], and only one relatively recent study [17], published in 2015, concerning the area of Brasov, in Central Romania.

In previously published studies from Romania, the most frequently isolated dermatophytes were *Trichophyton mentagrophytes* var. *interdigitale* (tinea pedis), *Trichophyton rubrum* (onychomycosis), *Microsporum audouinii,* and *Trichophyton violaceum* (tinea capitis); *M. audouinii* was isolated “mainly from epidemic foci” [14,15,16,17,18]. According to the 2015 study on human dermatophytosis in the area of Brasov [17], the ranking of the identified dermatophytes species was as follows: *T*. *rubrum* (78.7%, tinea unguium, tinea pedis), *T. mentagrophytes* (10.6%, tinea unguium, tinea pedis), *Microsporum canis* (6.4%, tinea corporis, tinea faciei, tinea barbae).

Romania is located between Central and Southeastern Europe. The country has a temperate and continental climate, with four clearly distinguishable seasons succeeding each other during typical years (spring, summer, autumn, and winter). The ecology and etiology of dermatophytosis is a dynamic field, with clinically relevant changes occurring in the frequency ranking of different species. Therefore, compared to previous studies, which have been mostly performed more than four decades ago, the prevalence of different dermatophyte species might have suffered changes.

In Cluj-Napoca, Romania, only a clinical diagnosis is performed in many cases of dermatophytosis when clinical symptoms are suggestive for superficial fungal infections. In these cases, tissue samples are not collected. Dermatologists mention two main reasons for this approach: it takes up to 3–4 weeks for a complete laboratory result to return, and empirical treatments avoid supplementary costs.

Therefore, the aim of this study was to investigate and identify the dermatophytes isolated from superficial fungal infections in samples from Cluj-Napoca, Cluj County (North-Western Romania). To the best of our knowledge, this is the first such study conducted in North-Western Romania.

## 2. Materials and Methods

**Figure 1:**
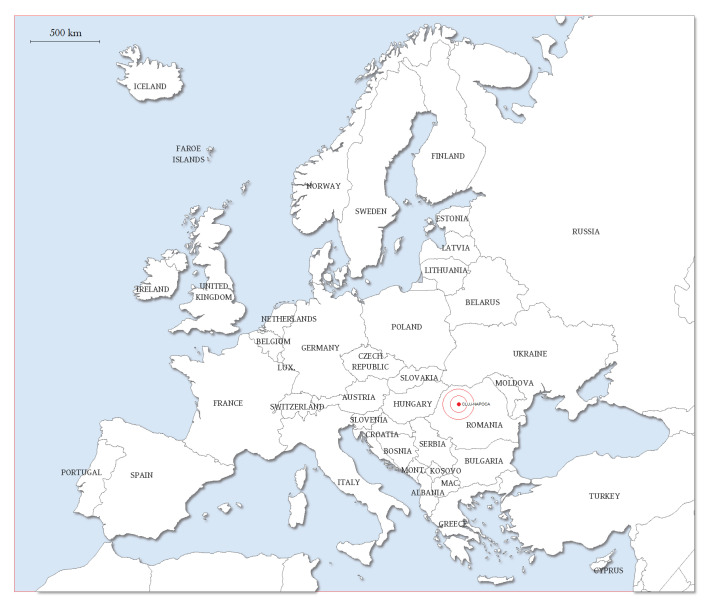
Location of Cluj-Napoca and North-Western Romania in Europe (map adapted from www.freeworldmaps.net).

Samples collected from outpatients with lesions suggestive for superficial fungal infections, who addressed 18 private practice dermatologists from Cluj-Napoca, Romania (Figure 1), were sent to the hospital laboratory of the Unirea Medical Center in Cluj-Napoca and identified at the Microbiology Department of the Iuliu Hațieganu University of Medicine and Pharmacy, Cluj-Napoca, Romania. Between May 2016 and April 2020, 350 samples (nails, skin, and hair) from 322 patients were examined.

All samples were examined by direct microscopy using KOH + 40% DMSO (dimethylsulphoxide) solution. The samples were cultured on Sabouraud chloramphenicol gentamicin agar slant and Sabouraud chloramphenicol gentamicin actidione agar slant (Bio-Rad, Marnes-la-Coquette, France). An incubation temperature of 27 °C was used and the cultures were examined twice every week for 3 weeks.

Dermathophytes and molds (non-dermathophyte filamentous fungi, NDF) were identified based on macroscopic and microscopic characteristics. For dermatophytes, a urea test (Urea indole medium, bioMérieux, Marcy l’Etoile, France) has also been performed. Non-dermathophyte filamentous fungi (NDF) were considered as etiological agents when fungal filaments were observed during direct microscopic examination of the samples and NDF were repeatedly isolated as pure culture from the majority of the inoculation points without concomitant isolation of a dermatophyte [19,20,21].

Yeasts were identified using chromogenic culture medium (Brilliance™ *Candida* Agar, Oxoid, Basingstoke, Hants, RG24 8PW UK), germ tube test, CANDIFAST^®^ (ELITech MICROBIO, Signes, France). Isolated yeasts were considered clinically relevant when the direct microscopic examination was positive (pseudo-filaments, numerous yeasts cells) and an abundant pure culture was detected [20,21].

This study presents a cross-sectional description of these laboratory data obtained between May 2016 and April 2020 by mycological diagnosis.

Data were collected and described using Microsoft Excel; confidence intervals were computed using StatPages (https://statpages.info, accessed on 25 April 2020).

The study was performed in accordance with the ethical standards of the 2013 revision of the Helsinki Declaration. Data obtained from biological samples was de-identified and analyzed anonymously, to eliminate the risk of a confidentiality breach occurring as part of this study. Ethical approval was waived by the local Ethics Committee of the Iuliu Hațieganu University of Medicine and Pharmacy from Cluj-Napoca, Romania, in view of the retrospective nature of the study and all the procedures being part of the routine care.

## 3. Results

The age of patients who addressed a dermatological private practice and for whom biological samples were collected for further investigation due to suspicions of superficial fungal infections ranged between 6 months and 88 years. The age distribution was as follows: 40 (12.4%) patients between 6 months and 15 years old; 97 (30.1%) patients between 16 and 30 years old; 117 (36.3%) patients between 31 and 45 years old; 46 (14.3%) patients between 46–60 years old; 22 (6.8%) patients >60 years old. Almost two thirds of the patients were female: 210 women (65.2%) and 112 men (34.8%).

Three hundred and fifty samples were collected: 22 (6.3%) hair samples, 69 (19.7) skin samples, and 259 (74%) nails samples.

**Figure 2:**
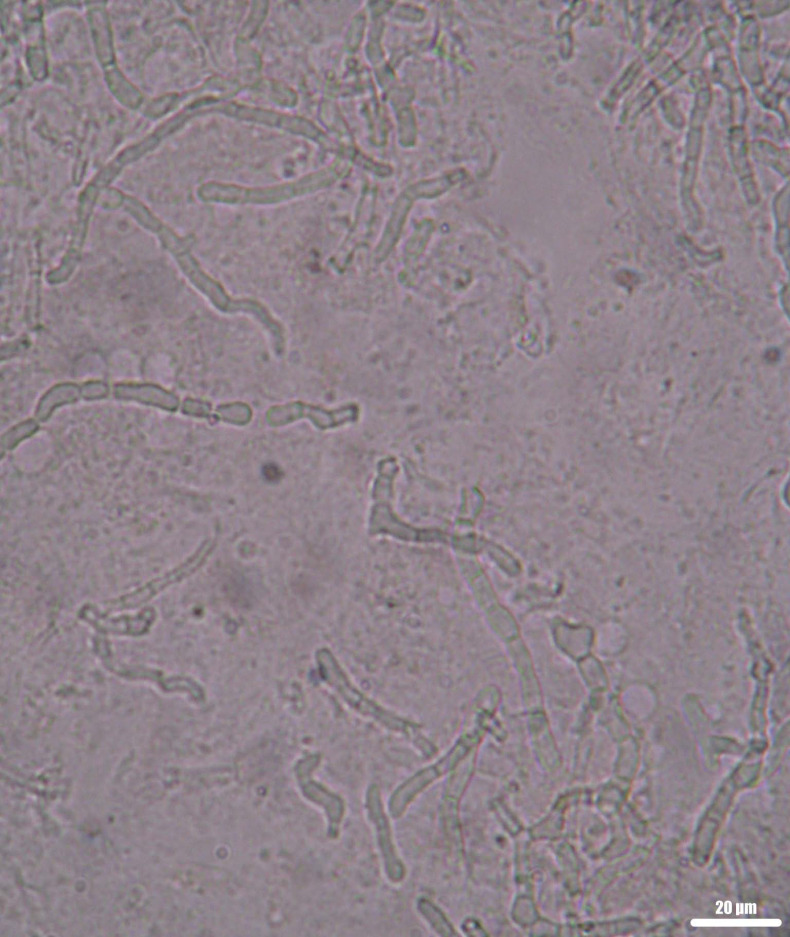
Septate fungal filaments in a clinical sample (nail). Wet mount, KOH + DMSO, direct microscopic examination, 400×.

One hundred samples, representing 28.6% (95% CI 23.9–33.6%) of the 350 collected samples were positive by either direct microscopy and/or culture (Figure 2).

Thirty-seven samples (10.6%) were positive by microscopy only; 13 samples (3.7%) were positive by culture only, and 50 samples (14.3%) were positive by culture and microscopy (Table 1).

These positive samples originated from 90 patients, representing 27.9% (95% CI 23.1–33.2%) of the 322 patients who were investigated for a clinical suspicion of dermatophytosis.

The positive predictive value (PPV) of a direct microscopy being confirmed by a positive culture was 57.5% (95% CI 49.8–63.4%). In 37 samples (10.6% of all samples), the microscopic examination was positive, but it could not be confirmed by a positive culture as well.

Thirteen of all 350 samples (3.7%) were negative by microscopy but positive by culture.

The negative predictive value (NPV) of a direct microscopy being confirmed by a negative culture was 95.06% (95% CI 92.5–97%), which corresponds to 250 negative cultures of 263 negative direct microscopic examinations.

Among the 63 (18%) positive cultures, 44 (69.8%) dermatophytes, two (3.2%) NDF, and 17 (27%) yeasts, *Candida albicans* and non-*albicans* were isolated. Fungal species isolated from the positive cultures according to the site of infection are presented in Table 2. Overall, among dermatophytes, the most frequently isolated species were *T. rubrum* (28/63, 44.4%) and *M. canis* (12/63, 19%).

The majority of the analyzed samples were nails and skin scales from the feet. *T. rubrum* was the most frequent dermathophyte (66.7%) isolated from infected toenails. The majority (60%) of the patients with toenail onychomycosis were males. *Candida* species (*C. albicans* and non-*albicans*) were the second etiological agent of toenail onychomycosis (26.7%), mostly isolated from women. In two cases of toenail infections, NDF were isolated: *Aspergillus* (3.3%) and *Fusarium* (3.3%).

All patients with tinea capitis were children, aged between 2 and 11 years. *M. canis* was isolated in 75% of the cases. Beside zoophilic species, anthropophilic dermatophytes were isolated from tinea capitis (Table 2): *Trichophyton tonsurans* (12.5%) and *T. rubrum* (12.5%). Only one case of tinea faciei has been identified and in which *M. canis* has been isolated.

*Candida* species (*C. albicans* and non-*albicans*) were isolated from almost 70% (9/13) of the samples collected from hands (skin or nails). The majority of the *Candida* species were isolated from women. Beside *Candida*, *T. rubrum* (4/13, 20%) was also isolated from fingernails and tinea manuum.

Zoophilic dermatophytes were the dominant species (66.7%) responsible for tinea corporis. *M. canis* was isolated from the majority (55.6%) of cases of tinea corporis (Table 2).

*Trichophyton mentagrophytes* was isolated in one case of tinea corporis from a patient with a history of animal contact. Anthropophilic dermatophytes, *T. rubrum* (22.2%) and *Trichophyton interdigitale* (11.1%), were also responsible for tinea corporis (Table 2). Most patients with tinea corporis were young, with ages ranging between 2 and 30 years.

## 4. Discussion

Superficial fungal infections of nails, hair, and skin are present, with varying prevalence, all over the world. Local socio-economic conditions and cultural practices, population mobility, changes in human lifestyle, local trauma, tight shoes, pets (cats, dogs, hamsters), familial transmission, sports (swimming, jogging, wrestling), are among the numerous risk factors identified for dermatophytoses, which can also influence their prevalence in a certain area [9,10,19].

There is currently a reasonable amount of data regarding dermatophytosis and dermatophytes available for most of the European countries, but little information from Romania [4,5,22,23,24]. Until 1952, *T. rubrum* represented only 2% of dermatophytes isolated in Romania, with constant increases during the next decades, until it became the main species responsible for onychomycosis [14,15,16,17]. This finding was also confirmed by our study, in which *T. rubrum* represented the most frequent species responsible for dermatophytosis. The results of the Achilles Project, a large-scale foot-screening project conducted in 16 European countries, as well as the results of numerous other studies showed *T. rubrum* as being the main species responsible for onychomycosis [3,4,25,26,27,28].

In the present study, *M. canis* (not *M. audouinii* or *T. violaceum)* was the dermatophyte most frequently isolated from tinea capitis, very likely because this dermatophytosis was found in family cases rather than in epidemic outbreaks among children collectivities [14]. Our findings were consistent with those of other studies [5,26,29], which reported *M. canis* as being the most common causative agent of tinea capitis in several European countries (Germany, Austria, Slovenia, Czech Republic, Italy, Greece). A survey regarding the etiology of tinea capitis conducted across 19 European countries showed that the main species was *M. canis*, but also reported an overall increase in the numbers of cases caused by *T. tonsurans*, an anthropophilic species [22].

In the current study only one case of tinea capitis by *T. tonsurans* was present. In contrast to our results, in other European countries (France, UK, the Netherlands) anthropophilic dermatophytes were the dominant agents in tinea capitis [23,25,30].

Beside *M. canis* and *T. tonsurans*, we isolated *T. rubrum* from one case of tinea capitis. This was an unusual finding, as *T. rubrum* only rarely infects beards, the hair, or the scalp [31,32].

*T. mentagrophytes* was isolated in one case of tinea corporis. Due to the constant decrease during the last three decades of the number of farm animals in Romanian villages and to the fact that most patients in the current study were from urban areas, the low prevalence (1.6%) of *T. mentagrophytes* was not surprising. In contrast to our low frequency, in several European studies *T. mentagrophytes* was isolated from skin and nails in higher rates, up to 23% [20,25,26,28].

In the current study, *T. interdigitale* was isolated only in 3.2% cases from tinea pedis and tinea corporis. In contrast to our data, in a recent study from Slovakia [33], *T. interdigitale* was found as an etiological agent in 10.6% of the cases (mainly tinea unguium, tinea pedis, and tinea inguinalis), being the second dermatophyte isolated, after *T. rubrum*. Other studies also found a higher frequency (9.5%) of *T. interdigitale,* isolated from feet (nails, skin) and fold samples [25].

Differentiating between *T. mentagrophytes* and *T. interdigitale* based only on morphological grounds (culture and microscopy) can be difficult. Species-specific identification is important for the management of patients, as it is able to also suggest the source of infection (anthropophilic, zoophilic, or geophilic). Although culture is still the only method able to confirm the viability of a fungus, molecular-based methods can accurately differentiate between close species, hence offering noteworthy information for the diagnosis, treatment, and case management [7,8].

In the present study, *Aspergillus* spp. and *Fusarium* spp. have been isolated each from one case of toenail onychomycosis, being responsible for 3.2% of positive cultures. Risk factors for NDF onychomycosis are represented by nail trauma, peripheral vascular diseases, advanced age, diabetes, contact with soil, and even the use of artificial nails. Other studies have isolated NDF from onychomycosis, especially from toenails, with various frequencies, widely ranging from 1.49% up to 33.2% [3,4,17,21,25,34,35,36].

Yeasts (*Candida* species) were isolated from infected nails (fingers and toenails), being the second (27%) etiological agent after *T. rubrum*, in this study. In the majority of cases, *Candida* was isolated from women. Other studies that found *Candida* as causative of onychomycosis, ranged its prevalence between 10% and 38.2% [3,4,6,25,36,37].

Despite exhibiting a high NPV of 95.06% (95% CI 92.5–97%), the low PPV (57.5%) of direct microscopy for being confirmed by a positive culture, and the fact that in 10.6% of all samples only the microscopic examination was positive, suggest the importance of performing both diagnostic methods and interpreting the results as part of their larger clinical context. This is especially true since using a molecular technique does not currently constitute a feasible option for the routine diagnosis of dermatophytosis.

In our study, only 13 of 350 (3.7%) samples were negative by microscopy but positive by culture. Differences between microscopic examination and culture were also found by other authors [2,25,26,38].

There are several possible explanations for the low positive rate of cultures among the positive microscopy samples found in our study (57.5%): the fungal filaments visible at microscopy were not viable; an already instituted treatment with antifungal drugs; an inadequate quantity of the collected sample; an incorrect sample collection. In superficial mycoses, laboratory diagnosis accuracy depends on the quality of samples [12,38,39,40]. Microscopic examination of clinical samples is an important tool, given the fact that in some cases the recovery of fungi from the samples cannot occur; in mycoses produced by molds or yeasts microscopic examination is an essential criterion for establishing their etiologic role [20,39].

Identification of a species implicated in superficial fungal mycoses is necessary for an adequate treatment and prophylactic measures. *Trichophyton* resistance to terbinafine appears to be an emerging problem [11]. For tinea capitis due to *M. canis*, griseofulvine is the recommended treatment with a response rate up to 88% [34,41,42]. Similar to other European countries, griseofulvine is no longer available in Romania, and therapeutic failure with terbinafine and itraconazole has been reported by authors from other countries [12,13,43].

### 4.1. Study Strengths

To the best of our knowledge, this is the first study conducted in North-Western Romania aiming to identify dermatophytes isolated from superficial fungal infections. By publishing this study, we hope to shed more light on the ecology, epidemiology, and etiology of dermatophytosis in Romania, for better management and treatment of patients with this type of pathology.

### 4.2. Study Limitations

A limitation of the current study was that only classical methods were used for the identification of the isolated fungi. Molecular methods are quicker, more sensitive and more specific compared to microscopy or culture alone for the identification of fungi, and could, therefore, represent a complementary option for the diagnostic of dermatophytosis. Molecular methods may also offer a reliable mycological diagnosis even in cases with low quality samples [8,11,38,39].

Currently, only few laboratories in Romania have the necessary equipment for performing molecular methods or MALDI-TOF mass spectrometry for routine diagnosis. Nevertheless, being confronted with the current SARS-CoV2 pandemic, a growing number of laboratories in Romania became operational for molecular diagnosis by purchasing adequate devices needed to enable this rapid and accurate type of diagnosis. We can only hope that the equipment will gradually also become available for the diagnosis of fungal infections.

Another limitation of the current study was the relatively low number of included tissue samples compared to other studies. To our knowledge, the laboratory where we performed this study is also the only one in the Cluj area to provide diagnosis through identification of dermatophytes (by microscopy and culture), hence the relatively low number of samples that could be included in this 4-year study (350 samples from 322 patients). Furthermore, many dermatologists do not collect tissue samples when clinical symptoms are suggestive for superficial fungal infections; therefore, only a clinical diagnosis is performed in many cases of dermatophytosis. The European Onychomycosis Observatory noted that about 60% of dermatologists do not perform sampling in cases of suspected dermatophytosis [24]. In their defense, dermatologists argued that in cases that are clinically suggestive for dermatophytosis it was not useful for the management of patients to wait for sometimes 3–4 weeks, until a positive culture (if any) confirmed and increased the precision of their diagnosis.

All these considerations emphasize the importance of using rapid, and highly sensitive and specific methods for the identification of fungi, which we also hope to be able to include in our future studies. Given the fact that it is difficult to draw solid conclusions based on small samples, we plan to continue this study as the number of investigated patients will increase over the following years.

Despite its limitations, this study updates and further contours the epidemiology and etiology of dermatophytosis in Romania, paving the way for a more effective management, treatment, and prevention of this type of pathology.

## 5. Conclusions

The main dermatophytes species identified from superficial fungal infections were *T. rubrum* and *M. canis*. *T. rubrum* was mostly isolated from onychomycoses, whereas *M. canis* was responsible for tinea capitis and tinea corporis in children. Yeasts (*Candida* species) were isolated from nails, especially from women.

Accurate diagnosis is essential for adequate treatment and prophylactic measures; hence, quicker and more accurate methods such as molecular methods or MALDI-TOF mass spectrometry should be used, whenever possible.

## Figures and Tables

**Table 1:** Results obtained after mycological examination of the samples (direct microscopic examination and culture).

Examination	Culture			Total
		+	−	
Direct microscopy	+	50	37	87
	−	13	250	263
Total		63	287	350

**Table 2:** Occurrence of dermatophytes, NDF (non-dermathophyte filamentous fungi) and yeasts isolated according to site of infection.

Fugal Species	Feet		Hands		Body/ Limbs *n*, %	Face *n*	Scalp, Hair *n*, %	Total *n*, %
	Toenails *n*, %	Skin *n*, %	Nails *n*, %	Skin *n*, %				
*T. rubrum*	20 (66.7)	1 (50)	2 (20)	2 (66.7)	2 (22.2)		1 (12.5)	28 (44.4)
*T. interdigitale*		1 (50)			1 (11.1)			2 (3.2)
*T. tonsurans*							1 (12.5)	1 (1.6)
*T. mentagrophytes*					1 (11.1)			1 (1.6)
*M. canis*					5 (55.6)	1	6 (75)	12 (19)
*Aspergillus* spp.	1 (3.3)							1 (1.6)
*Fusarium* spp.	1 (3.3)							1 (1.6)
Yeasts (*Candida*)	8 (26.7)		8 (80)	1 (33.3)				17 (27)
Total	30	2	10	3	9	1	8	63 (100)

*n* represents the number of isolated strains.

## References

[1] Ebrahimi M., Zarrinfar H., Naseri A., Najafzadeh M.J., Fata A., Parian M., Khorsand I., Novak Babič M. (2019). Epidemiology of dermatophytosis in northeastern Iran; a subtropical region. Curr. Med. Mycol..

[2] Silva-Rocha W.P., de Azevedo M.F., Chaves G.M. (2017). Epidemiology and fungal species distribution of superficial mycoses in Northeast Brazil. J. Mycol. Med..

[3] Sigurgeirsson B., Baran R. (2014). The prevalence of onychomycosis in the global population: A literature study. J. Eur. Acad. Dermatol. Venereol..

[4] Burzykowski T., Molenberghs G., Abeck D., Haneke E., Hay R., Katsambas A., Roseeuw D., van de Kerkhof P., van Aelst R., Marynissen G. (2003). High prevalence of foot diseases in Europe: Results of the Achilles Project. Mycoses.

[5] Ginter-Hanselmayer G., Weger W., Ilkit M., Smolle J. (2007). Epidemiology of tinea capitis in Europe: Current state and changing patterns. Mycoses.

[6] Foster K.W., Ghannoum M.A., Elewski B.E. (2004). Epidemiologic surveillance of cutaneous fungal infection in the United States from 1999 to 2002. J. Am. Acad. Dermatol..

[7] de Hoog G.S., Dukik K., Monod M., Packeu A., Stubbe D., Hendrickx M., Kupsch C., Stielow J.B., Freeke J., Göker M. (2017). Toward a novel multilocus phylogenetic taxonomy for the dermatophytes. Mycopathologia.

[8] Gräser Y., Saunte D.M.L. (2020). A hundred years of diagnosing superficial fungal infections: Where do we come from, where are we now and where would we like to go?. Acta Derm. Venereol..

[9] Havlickova B., Czaika V.A., Friedrich M. (2008). Epidemiological trends in skin mycoses worldwide. Mycoses.

[10] Zhan P., Liu W. (2017). The changing face of dermatophytic infections worldwide. Mycopathologia.

[11] Monod M., Méhul B. (2019). Recent findings in onychomycosis and their application for appropriate treatment. J. Fungi.

[12] Gupta A.K., Mays R.R., Versteeg S.G., Piraccini B.M., Shear N.H., Piguet V., Tosti A., Friedlander S.F. (2018). Tinea capitis in children: A systematic review of management. J. Eur. Acad. Dermatol. Venereol..

[13] Alkeswani A., Cantrell W., Elewski B. (2019). Treatment of tinea capitis. Skin Appendage Disord..

[14] Alteras I., Cojocaru I. (1968). Sur l’état actuel des dermatophytes en Roumanie. Mycopathologia et Mycologia Applicata.

[15] Alteras I. (1971). A short review on the onychomycosis by dermatophytes isolated in Bucharest. Mykosen.

[16] Alteras I., Cojocaru I. (1973). A short review on tinea pedis by dermatophytes. Mykosen.

[17] Irimie M., Oanta A., Irimie C.A., Minea D.I. (2015). Prevalence and antifungal susceptibility patterns of dermatophytes isolated from patients with neoplastic diseases: A case control study. Acta Dermatovenerol. Croat..

[18] Mareș M., Moroti-Constantinescu V.R., Denning D.W. (2018). The burden of fungal diseases in Romania. J. Fungi.

[19] Papini M., Piraccini B.M., Difonzo E., Brunoro A. (2015). Epidemiology of onychomycosis in Italy: Prevalence data and risk factor identification. Mycoses.

[20] Panasiti V., Devirgiliis V., Borroni R.G., Mancini M., Curzio M., Rossi M., Bottoni U., Calvieri S. (2007). Epidemiology of dermatophytic infections in Rome, Italy: A retrospective study from 2002 to 2004. Med. Mycol..

[21] Gupta A.K., Drummond-Main C., Cooper E.A., Brintnell W., Piraccini B.M., Tosti A. (2012). Systematic review of nondermatophyte mold onychomycosis: Diagnosis, clinical types, epidemiology, and treatment. J. Am. Acad. Dermatol..

[22] Hay R.J., Robles W., Midgley G., Moore M.K., European Confederation of Medical Mycology Working Party on Tinea Capitis (2001). Tinea capitis in Europe: New perspective on an old problem. J. Eur. Acad. Dermatol. Venereol..

[23] Fuller L.C. (2009). Changing face of tinea capitis in Europe. Curr. Opin. Infect Dis.

[24] Effendy I., Lecha M., Feuilhade de Chauvin M., Di Chiacchio N., Baran R., European Onychomycosis Observatory (2005). Epidemiology and clinical classification of onychomycosis. J. Eur. Acad. Dermatol. Venereol..

[25] Faure-Cognet O., Fricker-Hidalgo H., Pelloux H., Leccia M.T. (2016). Superficial fungal infections in a French teaching hospital in Grenoble area: Retrospective study on 5470 asmples from 2001 to 2011. Mycopathologia.

[26] Maraki S., Mavromanolaki V.E. (2016). Epidemiology of dermatophytoses in Crete, Greece: A 5-year survey. Med. Mycol. J..

[27] Svejgaard E.L., Nilsson J. (2004). Onychomycosis in Denmark: Prevalence of fungal nail infection in general practice. Mycoses.

[28] Saunte D.M., Svejgaard E.L., Haedersdal M., Frimodt-Møller N., Jensen A.M., Arendrup M.C. (2008). Laboratory-based survey of dermatophyte infections in Denmark over a 10-year period. Acta Derm. Venereol..

[29] Brajac I., Stojnić-Sosa L., Prpić L., Loncarek K., Gruber F. (2004). The epidemiology of *Microsporum canis* infections in Rijeka area, Croatia. Mycoses.

[30] Fuller L.C., Child F.C., Midgley G., Higgins E.M. (2003). Scalp ringworm in south-east London and an analysis of a cohort of patients from a paediatric dermatology department. Br. J. Dermatol..

[31] Abdel-Rahman S.M., Penny J., Alander S.W. (2004). *Trichophyton rubrum* tinea capitis in a young child. Pediatr. Dermatol..

[32] Larone D.H. (2002). Medically Important Fungi. A Guide to Identification.

[33] Baranová Z., Kampe T., Dorko E., Rimárová K. (2018). Epidemiological and clinical aspects of dermatophytoses in Eastern Slovakia: A retrospective three-year study. Cent. Eur. J. Public Health.

[34] Bonifaz A., Cruz-Aguilar P., Ponce R.M. (2007). Onychomycosis by molds. Report of 78 cases. Eur. J. Dermatol..

[35] Moreno G., Arenas R. (2010). Other fungi causing onychomycosis. Clin. Dermatol..

[36] Nouripour-Sisakht S., Mirhendi H., Shidfar M.R., Ahmadi B., Rezaei-Matehkolaei A., Geramishoar M., Zarei F., Jalalizand N. (2015). Aspergillus species as emerging causative agents of onychomycosis. J. Mycol. Med..

[37] Ja¨rv H., Naaber P., Kaur S., Eisen M., Silm H. (2004). Toenail onychomycosis in Estonia. Mycoses.

[38] Petinataud D., Berger S., Ferdynus C., Debourgogne A., Contet-Audonneau N., Machouart M. (2016). Optimising the diagnostic strategy for onychomycosis from sample collection to fungal identification evaluation of a diagnostic kit for real-time PCR. Mycoses.

[39] Pihet M., Le Govic Y. (2017). Reappraisal of conventional diagnosis for dermatophytes. Mycopathologia.

[40] Rudramurthy S.M., Shaw D. (2017). Overview and update on the laboratory diagnosis of dermatophytosis. Clin. Dermatol. Rev..

[41] Fuller L.C., Barton R.C., Mohd Mustapa M.F., Proudfoot L.E., Punjabi S.P., Higgins E.M. (2014). British Association of Dermatologists’ guidelines for the management of tinea capitis 2014. Br. J. Dermatol..

[42] Gupta A.K., Drummond-Main C. (2013). Meta-analysis of randomized, controlled trials comparing particular doses of griseofulvin and terbinafine for the treatment of tinea capitis. Pediatr. Dermatol..

[43] Kakourou T., Uksal U., European Society for Pediatric Dermatology (2010). Guidelines for the management of tinea capitis in children. Pediatr. Dermatol..

